# Supplementary material for: Paracrine control of α-cell glucagon exocytosis is compromised in human type-2 diabetes
Source: Nat Commun. 2020 Apr 20;11:1896. doi: 10.1038/s41467-020-15717-8 (PMC7171169; doi:10.1038/s41467-020-15717-8)
Supplement: Supplementary file 1 — Supplementary Information [file 41467_2020_15717_MOESM1_ESM.docx]

**Paracrine control of α-cell glucagon exocytosis is compromised in human type-2 diabetes**

**Omar-Hmeadi et al.**

# **Supplementary figures**

##
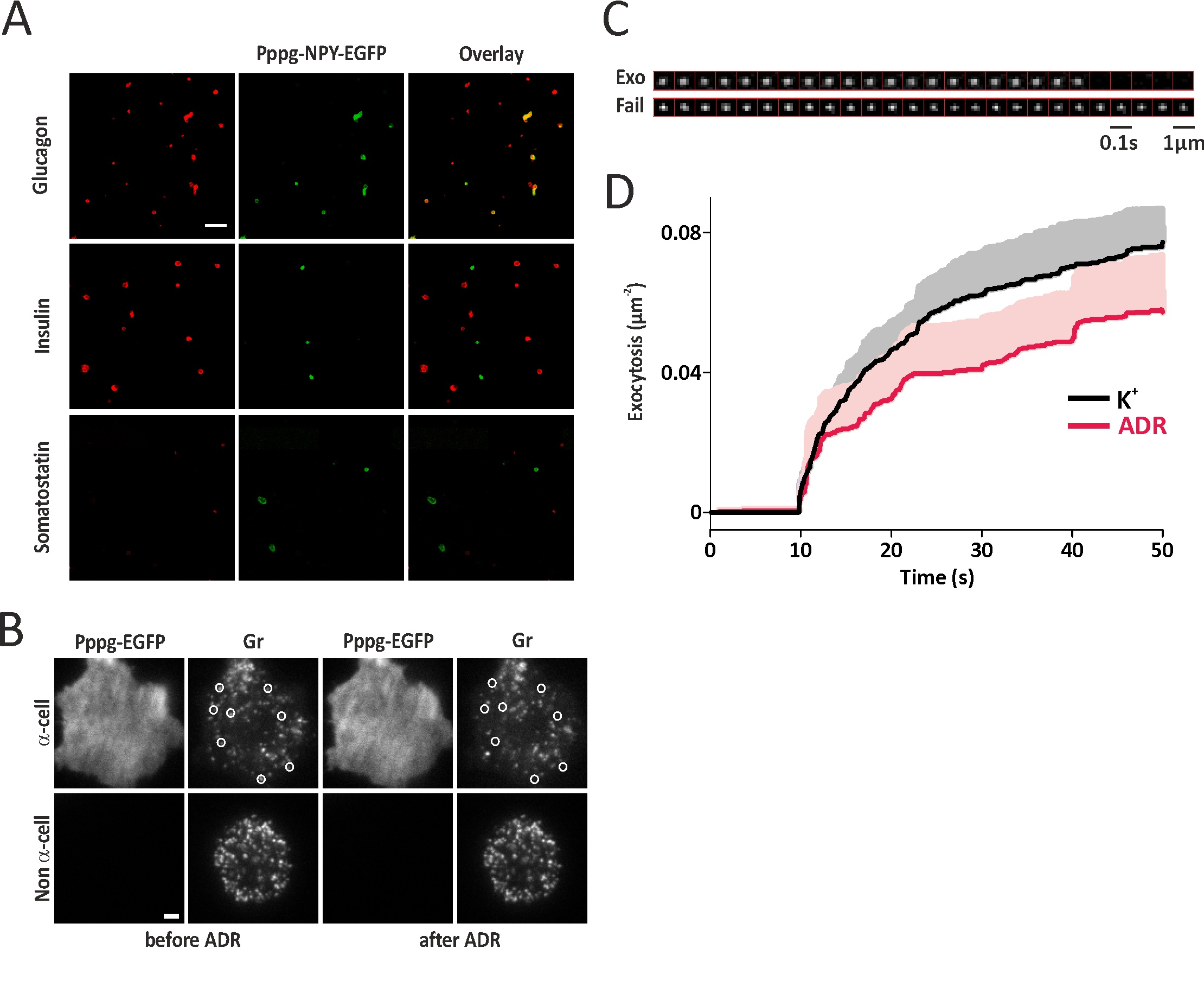


## **Supplementary Fig 1. Identification of human pancreatic α-cells.**

(**A**) Confocal images of Pppg-NPY-EGFP (green) expressing cells immunostained for glucagon (upper, red), insulin (middle, red) or somatostatin (lower, red) positive cells in a dispersed human islet α-cells. Scale bar 50 µm. Representative for 40 images.

(**B**) TIRF images of α-cells (top) expressing Pppg-EGFP together with NPY-Cherry and non α-cells (bottom) expressing NPY-Cherry before (left half) and after stimulation (right half) with 5µM adrenaline for 40s. Granules lost by exocytosis are highlighted by circles. Scale bar 2 µm.

(**C**) Image sequences showing example of granules undergoing exocytosis (exo, top) or failing to exocytose (fail, bottom) during stimulation of cells as in (**B**). Images are 0.1s apart.

(**D**) Average timecourse of exocytosis (cumulative event count normalized by cell footprint area, mean+/-SEM) in adrenaline stimulation experiments (pink) or K^+^ stimulation experiments (black). Stimulation lasted from 10-50s.

##
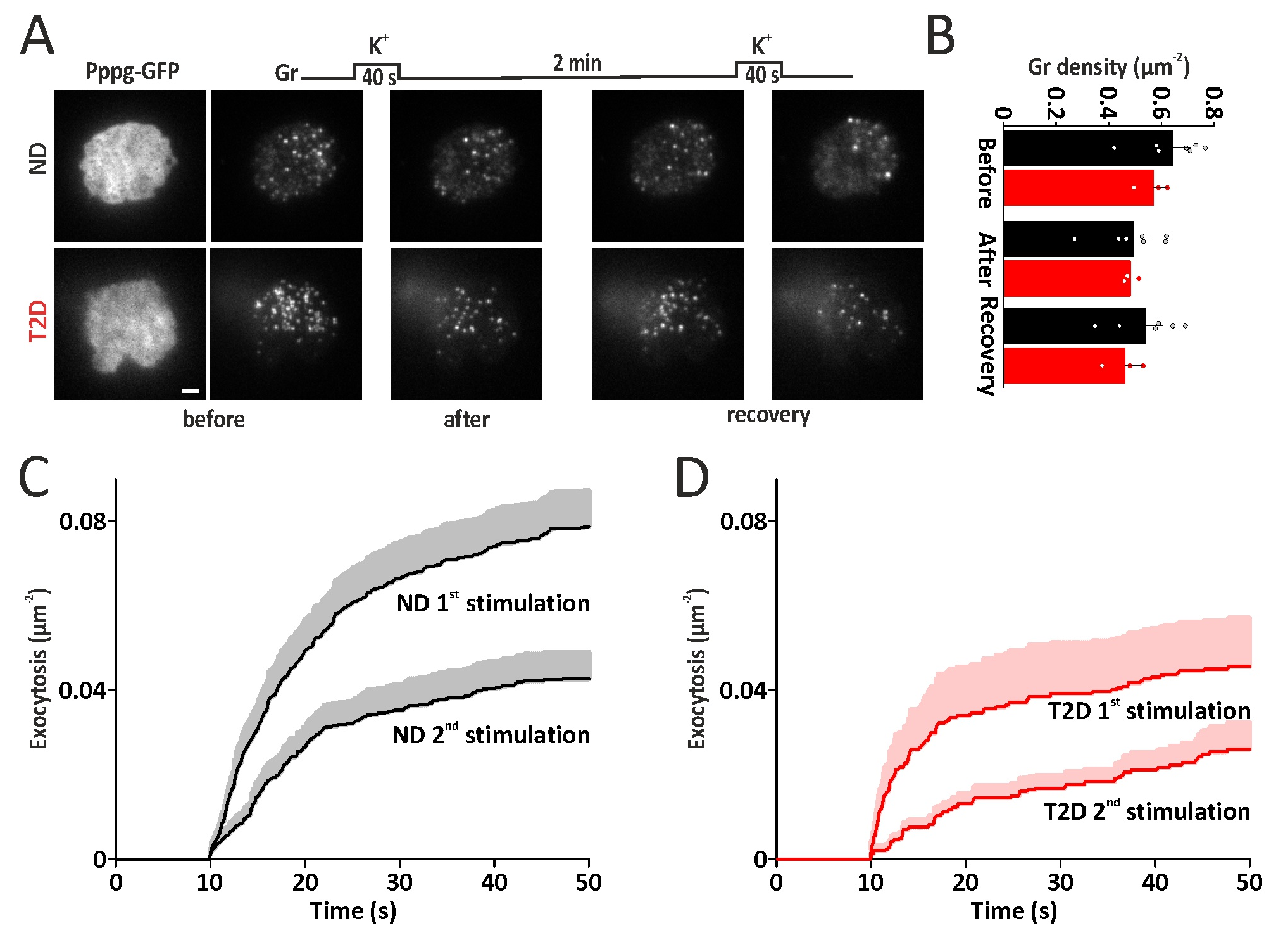


## **Supplementary Fig 2. Depletion and recovery of docked granules during K^+^ induced exocytosis**

(**A**) Examples of ND (top, representative for 7 donors) and T2D α-cell (bottom, representative for 3 donors) at indicated times during the indicated dual pulse stimulation protocol. Scale bar 2 µm.

(**B**) Average granule density of cells as in A (ND, black; T2D, red) at times corresponding to those indicated in A. The initial density of docked granules was 0.62 gr*µm^-2^ in ND and 0.57 gr*µm^-2^ in T2D α-cells (p=NS; n=7 ND vs n=3 T2D donors with 5-15 cells each). The first depolarization released ~15% of the docked granules in T2D α-cells (n=17) and 24 % of the docked granules in ND cells (n=55; p<n.s by two-tailed t-test). After the 2 min resting period, recovery of docked granules was negligible in T2D α-cells, compared with 8% in ND α-cells. Data are presented as mean values ± SEM.

(**C-D**) Average time course of cumulative exocytosis (normalized event count, mean+/-SEM) in ND (C) and T2D (D) experiments as in (A), for first and second stimulation.


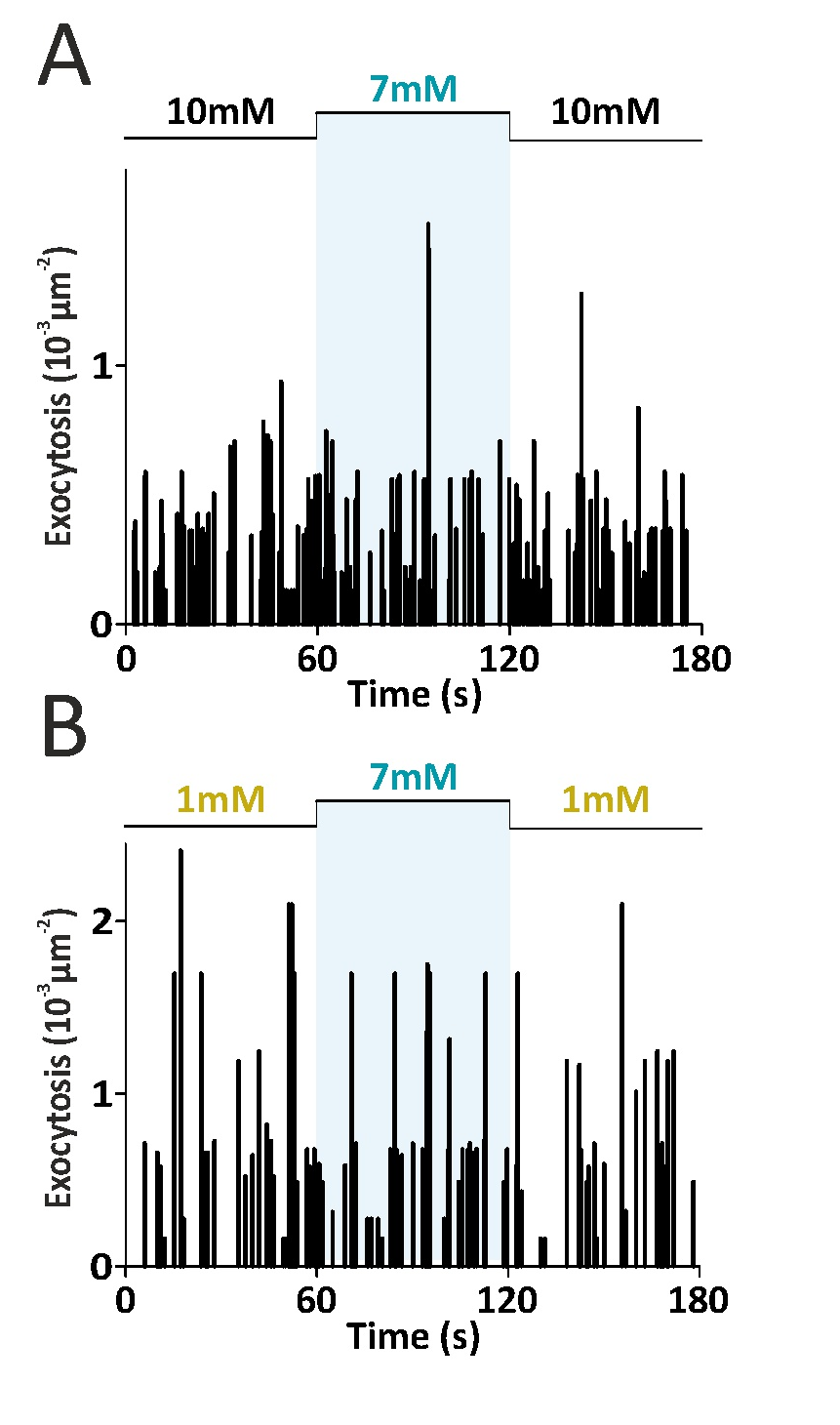


**Supplementary Fig 3. Acute response of α-cell exocytosis to change in glucose**

(**A-B**) Time course of exocytosis frequency in ND α-cells challenged with changes in glucose, from 10 to 7 mM and back (A) or from 1 to 7mM and back (B), as indicated.


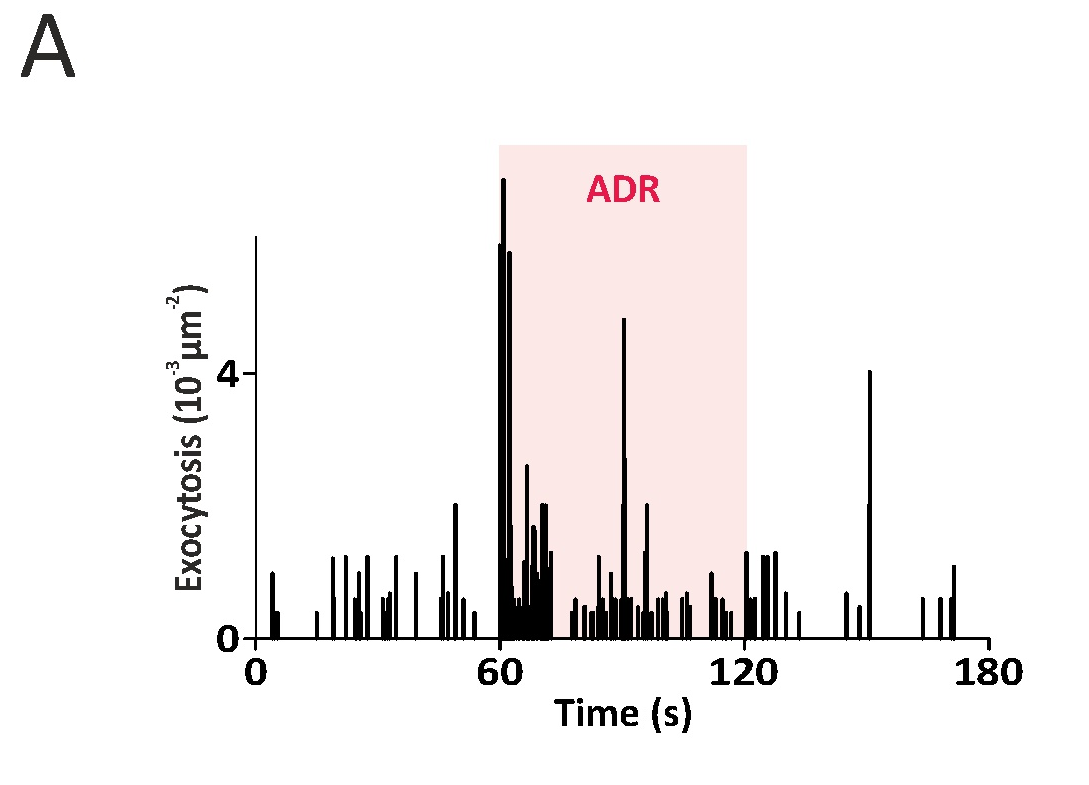


**Supplementary Fig 4. Regulation of glucagon exocytosis by insulin and adrenaline.**

(**A**) Time course of exocytosis frequency in ND α-cells challenged with adrenalin (ADR, as indicated), in presence of 10mM glucose.


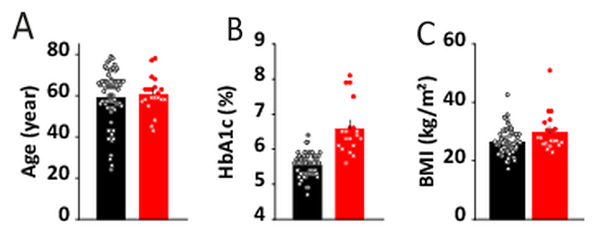


**Supplementary Fig 5. Human donor information.**

Distributions of (**A**) Age, (**B**) HbA1c, and (**C**) BMI for non-diabetic (black) and type-2 diabetic donors (red). The Isolation-IDs were: **Non-diabetic Islets** (42 male, 26 female). Uppsala: 2088, 2089, 2102, 2107, 2109, 2112, 2114, 2119, 2132, 2150, 2152, 2154, 2155, 2157, 2164, 2169, 2170, 2172, 2176, 2185, 2188, 2191, 2193, 2194, 2195, 2205, 2207 2209, 2217, 2218, 2225, 2228, 2232, 2233, 2245, 2246, 2247, 2253, 2254, 2255, 2258, 2275, 2278, 2295, 2298, 2301, 2309, 2310, 2317, 2326, 2327, 2334, 2337, 2344, 2346, 2389, 2398, 2400, 2402, 2403, 2431, 2460, 2467, 2472, 2478; Edmonton: R253, R249, R290; **ND Sections** (2 male, 3 female): Uppsala: 1396, 1484, 1784, 1921, 2108. **Type-2 diabetic Islets** (13 male, 8 female). Uppsala: 2133, 2151, 2199, 2202, 2203, 2211, 2219, 2238, 2240, 2274, 2299, 2350, 2395, 2438, 2465, 2471; Edmonton: R244, R192, R259, R307, R347. **T2D Sections** (1 male, 4 female), Uppsala: 1857, 1864, 1923, 2061, 2211.

**KEY RESOURCES TABLE**

| REAGENT or RESOURCE | SOURCE | IDENTIFIER |
| --- | --- | --- |
| **Antibodies** | | |
| Mouse anti-Glucagon (K79bB10) , dil 1/1500 | Sigma | G2654 |
| Rabbit anti-Glugacon , dilution 1/200 | DAKO (Uppsala) | A0565 |
| Guinea Pig anti-Insulin , dilution 1/200 | DAKO (Uppsala) | A0564 |
| Rabbit anti-Somatostatin , dilution 1/200 | DAKO (Uppsala) | A0566 |
| Rabbit anti-SSTR2 (UMB1), , dilution 1/100 | abcam | ab134152 |
| Goat anti-Rabbit Alexa-546, dilution 1/500 | Invitrogen | A11035 |
| Goat anti-Mouse Alexa-555, dilution 1/500 | Invitrogen | A21424 |
| Goat anti-Guinea Pig Alexa-555, dil 1/500 | Invitrogen | A21435 |
| Goat anti-Rabbit Alexa-488, dilution 1/500 | Invitrogen | A11034 |
| **Bacterial and Virus Strains** | | |
| Adenovirus coding for NPY-mCherry | P Rorsman (Oxford) | n/a |
| Adenovirus coding for NPY-Td-Orange | This paper |  |
| Adenovirus coding for Pppg-GFP | A Tengholm (Uppsala) | n/a |
| Adenovirus coding for Pppg-NPY-GFP | This paper |  |
| **Biological Samples** |  |  |
| Human pancreatic islets (Goto et al., 2004) | Nordic Network for Clinical Islet Transplantation Uppsala |  |
| Human pancreatic islets (Lyon et al., 2016) | ADI Isletcore, University of Alberta (Edmonton) |  |
| **Chemicals, Peptides, and Recombinant Proteins** | | |
| Cell dissociation buffer | Thermo Fisher | 13150016 |
| Trypsin solution | Thermo Fisher | 12604-021 |
| Lipofectamine 3000 | Thermo Fisher | L3000015 |
| Polylysine | Sigma-Aldrich | P5899 |
| CMRL media | Thermo Fisher | 21530027 |
| L-Glutamine | Hyclone | SH30034.01 |
| Background sniper | Biocare Medical | BS966 |
| Dako wash baffer | Dako | S3006 |
| insulin | Sigma-Aldrich | I1507 |
| Forskolin | Sigma-Aldrich | F6886 |
| Somatostatin | Sigma-Aldrich | S9129 |
| Polylysine | Sigma-Aldrich | P5899 |
| GABA | Sigma-Aldrich | A2129 |
| adrenaline | Sigma-Aldrich | Y0000882 |
| Doxycycline | Sigma-Aldrich | D9891 |
| **Software and Algorithms** | | |
| MetaMorph | Molecular Devices | RRID:SCR_002368 |
| ImageJ | imagej.nih.gov |  |
| Origin 2018 | originlab.com |  |
| PatchMaster | heka.com |  |
