# Supplementary material for: Paracrine control of α-cell glucagon exocytosis is compromised in human type-2 diabetes
Source: Nat Commun. 2020 Apr 20;11:1896. doi: 10.1038/s41467-020-15717-8 (PMC7171169; doi:10.1038/s41467-020-15717-8)
Supplement: Supplementary file 3 — Reporting Summary [file 41467_2020_15717_MOESM3_ESM.pdf]

Corresponding author(s): Sebastian Barg
Last updated by author(s): Mar 02, 2020

nature research

Reporting Summary

Nature Research wishes to improve the reproducibility of the work that we publish. This form provides structure for consistency and transparency in reporting. For further information on Nature Research policies, see Authors & Referees and the Editorial Policy Checklist .

Statistics

For all statistical analyses, confirm that the following items are present in the figure legend, table legend, main text, or Methods section.

n/a	Confirmed	
[ ]	[x]	The exact sample size (*n*) for each experimental group/condition, given as a discrete number and unit of measurement
[ ]	[x]	A statement on whether measurements were taken from distinct samples or whether the same sample was measured repeatedly
[ ]	[x]	The statistical test(s) used AND whether they are one- or two-sided<br>*Only common tests should be described solely by name; describe more complex techniques in the Methods section.*
[x]	[ ]	A description of all covariates tested
[x]	[ ]	A description of any assumptions or corrections, such as tests of normality and adjustment for multiple comparisons
[ ]	[x]	A full description of the statistical parameters including central tendency (e.g. means) or other basic estimates (e.g. regression coefficient) AND variation (e.g. standard deviation) or associated estimates of uncertainty (e.g. confidence intervals)
[ ]	[x]	For null hypothesis testing, the test statistic (e.g. *F*, *t*, *r*) with confidence intervals, effect sizes, degrees of freedom and *P* value noted<br>*Give P values as exact values whenever suitable.*
[x]	[ ]	For Bayesian analysis, information on the choice of priors and Markov chain Monte Carlo settings
[x]	[ ]	For hierarchical and complex designs, identification of the appropriate level for tests and full reporting of outcomes
[x]	[ ]	Estimates of effect sizes (e.g. Cohen's *d*, Pearson's *r*), indicating how they were calculated

Our web collection on statistics for biologists contains articles on many of the points above.

Software and code

Policy information about availability of computer code

Data collection	Imaging: MetaMorph v7, Electrophysiology: HEKA Patchmaster v2
Data analysis	MetaMorph v7, ImageJ v1.52 with custom analysis routines (Gandasi Nat Comm 2014), Origin Pro v2018

For manuscripts utilizing custom algorithms or software that are central to the research but not yet described in published literature, software must be made available to editors/reviewers. We strongly encourage code deposition in a community repository (e.g. GitHub). See the Nature Research guidelines for submitting code & software for further information.

Data

Policy information about availability of data

All manuscripts must include a data availability statement. This statement should provide the following information, where applicable:

- Accession codes, unique identifiers, or web links for publicly available datasets
- A list of figures that have associated raw data
- A description of any restrictions on data availability

We have no restrictions on sharing the raw data. However, we are not aware of a practical way to make the raw data (600GB) available through public repositories.

Field-specific reporting

Please select the one below that is the best fit for your research. If you are not sure, read the appropriate sections before making your selection.

[x] Life sciences   [ ] Behavioural & social sciences   [ ] Ecological, evolutionary & environmental sciences

For a reference copy of the document with all sections, see nature.com/documents/nr-reporting-summary-flat.pdf

Life sciences study design

All studies must disclose on these points even when the disclosure is negative.

Sample size	No sample size calculation was performed, and sample size was instead based on previous experience with single cells and human islets (Gandasi et al Cell Metab 2018 DOI: 10.1016/j.cmet.2017.12.017).
Data exclusions	No data were excluded.
Replication	At least 2 biological repetitions for each experiment, meaning islets from different donors on different days. All repetitions at repetition were successful and included in the statistical analysis.
Randomization	Not relevant. When comparing eg drug effects, islet samples were divided onto several coverslips that were treated differently only during data acquisition.
Blinding	Blinding was not formally used, but data files from individual donors were processed together and without knowledge of the treatment group (except for diabetic status, which was known on arrival of islet samples).

Reporting for specific materials, systems and methods

We require information from authors about some types of materials, experimental systems and methods used in many studies. Here, indicate whether each material, system or method listed is relevant to your study. If you are not sure if a list item applies to your research, read the appropriate section before selecting a response.

Materials & experimental systems

n/a	Involved in the study	
☐	☒	Antibodies
☒	☐	Eukaryotic cell lines
☒	☐	Palaeontology
☒	☐	Animals and other organisms
☐	☒	Human research participants
☒	☐	Clinical data

Methods

n/a	Involved in the study	
☒	☐	ChIP-seq
☒	☐	Flow cytometry
☒	☐	MRI-based neuroimaging

Antibodies

Antibodies used

Mouse anti-Glucagon (K79bB10) Sigma G2654, dilution 1/1500
Rabbit anti-Glugacon DAKO (Uppsala) A0565, dilution 1/200
Guinea Pig anti-Insulin DAKO (Uppsala) A0564, dilution 1/200
Rabbit anti-Somatostatin DAKO (Uppsala) A0566, dilution 1/200
Rabbit anti-SSTR2 (UMB1) abcam ab134152, dilution 1/100

Goat anti-Rabbit Alexa-546 Invitrogen A11035, dilution 1/500
Goat anti-Mouse Alexa-555 Invitrogen A21424, dilution 1/500
Goat anti-Guinea Pig Alexa-555 Invitrogen A21435, dilution 1/500
Goat anti-Rabbit Alexa-488 Invitrogen A11034, dilution 1/500

Validation

All primary antibodies are tested and characterized as specific in human tissues, by the manufacturers, and are widely cited.

Mouse anti-Glucagon (Sigma G2654): IHC in fixed sections of pancreas from human, www.sigmaaldrich.com/catalog/product/sigma/g2654, antibodyregistry.org/AB_259852

anti-glucagon (DAKO A0565): antibodyregistry.org/AB_10013726, IHC: www.proteinatlas.org/ENSG00000115263-GCG/antibody

anti-insulin (DAKO A0564): antibodyregistry.org/AB_10013624, www.citeab.com/antibodies/3382917-a0564-insulin

anti-somatostatin (DAKO A0566): antibodyregistry.org/AB_2688022, IHC: www.proteinatlas.org/ENSG00000157005-SST/antibody

anti SSTR2 (abcam ab134152): manufacturer information: reacts with mouse, rat, human, tested in human pancreas and SSTR2 ko mice. Fischer et al Clin Endocrinol Metab 93:4519-24 (2008), antibodyregistry.org/ab134152

Human research participants

Policy information about studies involving human research participants

Population characteristics	Islets were from 68 non-diabetic donors (42 male, 26 female, age 59.0+/-12.9, BMI 26.5+/-4.3, HbA1c 5.5+/-0.3; mean+/-SD) and 21 type-2 diabetic donors (13 male, 8 female, age 60.4+/-8.7, BMI 29.7+/-6.3, HbA1c 6.6+/-0.7; mean+/-SD).<br><br>Sections were from 5 ND (2 male, 3 female, age 63.8+/-6.2, BMI 25.4+/-4.9, HbA1c 6.3+/-2.1) and 5 type-2 diabetic donors (1 male, 4 female, age 62.4+/-5.9, BMI 33.9+/-2.2, HbA1c 7.6+/-0.4; mean+/-SD).
Recruitment	Cadaveric donor pancreata deemed unsuitable for transplantation were obtained and processed by the islet isolation cores. No selection criteria were applied.
Ethics oversight	Human pancreatic islets and pancreas sections were obtained from the Nordic Network for Clinical Islet Transplantation Uppsala (ethical approved by Uppsala Regional Ethics Board) or the ADI Isletcore at the University of Alberta (ethical approval by Alberta Human Research Ethics Board, Pro00001754) and with the donor families' written informed consent. The study was approved by the Uppsala Regional Ethics Board (2006/348).

Note that full information on the approval of the study protocol must also be provided in the manuscript.
